# Methicillin-resistant *Staphylococcus aureus* bacteremia with elevated vancomycin minimum inhibitory concentrations

**DOI:** 10.1017/ash.2023.163

**Published:** 2023-05-02

**Authors:** Alexandra G. Mills, Amy C. Dupper, Kieran I. Chacko, Bremy Alburquerque, Devika Nadkarni, Ana Berbel Caban, Lindsey Fox, Melissa R. Gitman, Ajay Obla, Harm van Bakel, Deena R. Altman

**Affiliations:** 1 Division of Infectious Diseases, Department of Medicine, Icahn School of Medicine at Mount Sinai, New York, New York; 2 Department of Genetics and Genomics Sciences, Icahn School of Medicine at Mount Sinai, New York, New York; 3 Department of Pathology, Molecular and Cell-Based Medicine, Icahn School of Medicine at Mount Sinai, New York, New York; 4 Department of Microbiology, Icahn School of Medicine at Mount Sinai, New York, New York; 5 Icahn Genomics Institute, Icahn School of Medicine at Mount Sinai, New York, New York

## Abstract

This case–control study of 25 cases with methicillin-resistant *Staphylococcus aureus* (MRSA) bacteremia with vancomycin minimum inhibitory concentration (MIC) ≥ 2 µg/mL and 391 controls (MIC < 2 µg/mL) characterized the clinical characteristics, treatments, and outcomes associated with elevated vancomycin MIC. Elevated vancomycin MIC was associated with baseline hemodialysis, prior MRSA colonization, and metastatic infection.

Bloodstream infection (BSI) with *Staphylococcus aureus* is associated with morbidity and mortality, posing significant management challenges.^1^ Although vancomycin remains the standard of care for methicillin-resistant *S. aureus* (MRSA) BSI, treatment failures do occur, often associated with elevated vancomycin minimum inhibitory concentrations (MICs).^1,2^ Although MRSA isolates with vancomycin MIC equal to 2 µg/mL are considered susceptible to vancomycin according to Clinical Lab and Standards Institute (CLSI) breakpoints,^3^ they have been associated with treatment failure despite susceptibility.^1^ Known risk factors for elevated MIC include prior vancomycin exposure, prolonged duration of bacteremia, and lack of source control.^2,4^

Management of MRSA BSI with elevated vancomycin MIC is not clearly defined.^1^ Although source control is central to management, decisions to continue vancomycin, switch to alternative monotherapies, or attempt salvage combination therapy are made on a case-by-case basis with variability in practices.^1,5^ Decisions are often multidisciplinary, between frontline providers, infectious diseases (ID) consultants, and ID pharmacists, along with established antimicrobial stewardship programs.

In this case–control study, we investigated risk factors and outcomes between those who do and do not develop MRSA BSI with vancomycin MIC ≥ 2 µg/mL. Furthermore, we have described the treatments chosen by providers in this challenging situation.

## Methods

This retrospective case–control study included adults (≥18 years) admitted to a quaternary care hospital in New York City with MRSA BSI over a 5-year period (2014–2019). The Clinical Microbiology Laboratory utilized matrix-assisted laser desorption/ionization-time of flight mass spectrometry (MALDI-TOF MS; Bruker Daltonics, Billerica, MA) for identification and Vitek 2 (bioMérieux) for susceptibilities. MICs of ≥ 2 µg/mL per Vitek 2 were verified by Etest (bioMérieux). Vancomycin MICs and interpretations were reported in the electronic medical record (EMR), with retesting of serially positive blood cultures every 72 hours.

Demographic and clinical data were extracted from the EMR for 416 consecutive adults (≥18 years) with MRSA BSI using previously described tools.^6^ Colonization was determined by positive MRSA culture prior to BSI, or documented history of MRSA colonization; MRSA swab screening was not routinely performed.

Due to the exploratory nature of this study, we performed univariable logistic regression with the outcome defined as MRSA BSI with a vancomycin MIC ≥ 2 µg/mL. Cases were adults (aged >18 years) with MRSA BSI with a vancomycin MIC ≥ 2 µg/mL, and controls were adults (aged >18 years) with MRSA BSI with a vancomycin MIC < 2 µg/mL. For controls, duration of bacteremia was measured from the first date of positive blood culture until the first date of negative blood culture. Duration of bacteremia is associated with elevated vancomycin MIC; thus, we calculated the date of first positive blood culture until the date of vancomycin MIC ≥ 2 µg/mL for all cases. We also performed univariable logistic regression on outcome variables with being a case or control as a predictor. A *P*-value approximating *P* ≤ .05 was considered notable for potential significance. Analyses were performed in R version 4.0.4 software.^7^

This study was approved by the institutional review board of the Icahn School of Medicine at Mount Sinai (protocol nos. 13-00981 and 17-00825). This study was performed in accordance with the Helsinki Declaration of 1964 and its later amendments.

## Results

Over the 5-year period (2014–2019), 25 (6%) of 416 of adults with MRSA BSI had an isolate with a vancomycin MIC ≥ 2 µg/mL. Overall, 9 patients had an MIC ≥ 2 µg/mL on initial cultures. Among them, 4 patients were transferred from other hospitals; thus, culture information was not available. All 25 cases were previously exposed to vancomycin.

Cases had higher odds of baseline hemodialysis (odds ratio [OR], 3.61; 95% confidence interval [CI], 1.55–8.40; *P* = .003) and a history of MRSA colonization (OR, 5.87; 95% CI, 2.29–15.05; *P <* .001) (Table 1). Although only approaching significance, cases had higher odds of hospital admission in the prior 90 days (OR, 2.80; 95% CI, 0.94–8.32; *P* = .06). Ability to achieve source control did not differ between the groups. The frequency of elevated vancomycin MIC did not increase over the study period.

**Table 1.** Univariable Analysis of Demographics and Clinical Characteristics of Cases with MRSA BSI with MIC ≥ 2 µg/mL Compared to Controls

Characteristic	Cases (n = 25), No. (%)	Controls (n = 391), No. (%)	Odds Ratio	95% Confidence Interval	*P* Value
**Sex**					
Male	15 (60)	245 (63)	0.89	0.39–2.04	.79
Female	10 (40)	146 (37)	Reference		
**Age at infection**					
18–54 y	9 (36)	124 (32)	Reference		
55–69 y	9 (36)	121 (31)	1.02	0.39–2.67	.96
≥70 y	7 (28)	146 (37)	0.66	0.24–1.83	.42
**Race/Ethnicity**					
Non-Hispanic White	9 (36)	155 (40)	Reference		
Non-Hispanic Black	7 (28)	100 (26)	1.21	0.44–3.34	.72
Hispanic	7 (28)	93 (24)	1.30	0.47–3.60	.62
Other	2 (8)	37 (10)	0.93	0.19–4.49	.93
Missing/Unknown	0	6			
**History of injection drug use**					
Yes	4 (20)	39 (10)	2.26	0.80–6.35	.12
No	20 (80)	352 (90)	Reference		
**Body mass index, kg/m** ^2^					
<18.5	1 (4)	49 (13)	0.32	0.03–2.55	.28
18.5–24.9	9 (36)	139 (36)	Reference		
25.0–29.9	8 (32)	94 (24)	1.1	0.49–3.53	.59
≥30.0	7 (28)	107 (28)	1.01	0.36–2.80	.98
Missing	0	2			
**HIV diagnosis**					
Yes	4 (16)	35 (9)	1.94	0.63–5.96	.25
No	21 (84)	356 (91)	Reference		
**Admission source**					
Home^a^	15 (60)	237 (61)	Reference		
NH/rehab/LTACH	3 (12)	102 (26)	0.4	0.13–1.6	.23
Outside hospital	7 (28)	52 (13)	2.13	0.83–5.48	.12
**Prior hospital admission (90 d)**					
Yes	21 (84)	255 (65)	2.80	0.94–8.32	.06
No	4 (16)	136 (35)	Reference		
**Length of hospital stay prior to BSI**					
CO-MRSA (≤ 3 days)	14 (5)	249 (64)	Reference		
HO-MRSA (> 3 days)	11 (44)	142 (36)	1.38	0.61–3.12	.44
**Hemodialysis**					
Yes	10 (40)	61 (16)	3.61	1.55–8.40	.003
No	15 (60)	330 (84)	Reference		
**Presence of invasive device^b^**					
Yes	22 (88)	286 (73)	2.69	0.79–9.18	.11
No	3 (12)	105 (27)	Reference		
**Invasive procedures^c^**					
Yes	13 (52)	165 (42)	1.48	0.66–3.34	.34
No	12 (48)	226 (58)	Reference		
**Wound present^d^**					
Yes	14 (56)	216 (55)	1.03	0.46–2.33	.94
No	11 (44)	175 (45)	Reference		
**Charlson comorbidity index^e^**					
0–3	7 (28)	112 (29)	Reference		
4–5	3 (12)	90 (23)	0.53	0.13–2.12	.37
6–8	9 (36)	120 (31)	1.20	0.43–3.33	.73
>8	6 (24)	69 (18)	1.39	0.45–4.31	.57
**History of transplant^f^**					
Yes	3 (12)	49 (13)	0.95	0.27–3.30	.94
No	22 (88)	342 (87)	Reference		
**History of MRSA colonization^g^**					
Yes	19 (76)	137 (35)	5.87	2.29–15.05	<.001
No	6 (24)	254 (65)	Reference		
**Presumed source of MRSA BSI**					
Skin infection	7 (28)	136 (35)	0.73	0.30–1.79	.49
Pneumonia	1 (4)	42 (11)	0.35	0.05–2.63	.31
Vascular access	9 (36)	103 (26)	1.57	0.67–3.67	.30
Other^h^	7 (28)	115 (29)	0.93	0.38–2.29	.88
**Bacteremic duration, median (IQR)** ^i^	5.0 (0.0– 13.0)	3.0 (1.0–5.0)	1.10	1.04–1.16	< .001
**Polymicrobial**					
Yes	3 (12)	44 (11)	1.08	0.31–3.74	.91
No	22 (88)	347 (89)	Reference		
**ICU admission prior to BSI**					
Yes	7 (32)	64 (17)	1.99	0.80–4.95	.14
No	18 (68)	327 (83)	Reference		
**Intubated prior to BSI**					
Yes	7 (28)	72 (18)	1.72	0.69–4.28	.24
No	18 (72)	319 (82)	Reference		

Note. BSI, bloodstream infection; HIV, human immunodeficiency virus; NH, nursing home; LTACH, long-term acute care hospital; MRSA, methicillin-resistant *Staphylococcus aureus*. CO, community-onset; HO, hospital-onset; IQR, interquartile range; ICU, intensive care unit; MIC, minimum inhibitory concentration.

a Included nonmedical residences such as home, group homes, assisted living facilities, and homeless shelters.

b Included pacemaker, implantable cardioverter defibrillator (ICD), left ventricular-assist device (LVAD), vascular access (excluding peripheral intravenous catheters), orthopedic hardware, nephrostomy, suprapubic catheter, ileal conduit, foley catheter, arteriovenous graft placement (AVG), percutaneous endoscopic gastrostomy (PEG) tube, or ostomy.

c Included any invasive procedures or surgery occurring within 1 month before first positive blood culture for MRSA, excluding electroencephalogram (EEG), electrocardiogram (EKG), and transthoracic echocardiogram (TTE).

d Included presence of a chronic skin wound overlying the sacrum, limb, abdomen, or other body part.

e Refer to standard definitions for CCI.

f Included solid organ and bone marrow transplant.

g Any positive culture from urine, sputum, tissue, or nares with MRSA prior to the positive MRSA blood culture or a documented history of prior MRSA infection or colonization.

h MRSA infection from urinary source, osteomyelitis, surgical site infection, spinal infection, septic arthritis or cardiac device infection, or an unknown/not reported source.

i Measured as time to development of elevated vancomycin MIC for cases and time to clearance of BSI in controls.

An outcome analysis revealed a 2.62 higher odds of recurrent MRSA BSI in cases (95% CI, 0.99–6.94; *P =* .05) (Table 2). Cases also had a 2.86 (95% CI, 1.23–6.61) higher odds of developing metastatic infection during their index admission for BSI (*P =* .01). Median time to discharge after initial positivity reached borderline significance at 34 days for cases (IQR, 15–47) compared with 15 days (IQR, 8–24) for controls (95% CI, 0.97–2.49; *P =* .07). Mortality rates did not differ.

**Table 2.** Outcomes of Cases with MRSA BSI with MIC ≥ 2 µg/mL Compared With Controls

Outcome	Cases (n = 25), No. (%)	Controls (n = 391), No. (%)	Odds Ratio	95% Confidence Intervals	*P* Value
**90-d mortality**					
Yes	7 (28)	116 (30)	0.92	0.37–2.27	.86
No	18 (72)	275 (70)	Reference		
**90-d mortality related to MRSA**					
Yes	4 (57)	77 (66)	0.68	0.14–3.17	.62
No	3 (43)	39 (34)	Reference		
Missing	18	275			
**> 50% rise in creatinine^a^**					
Yes	10 (63)	140 (41)	2.42	0.86–6.80	.09
No	6 (37)	203 (59)	Reference		
Missing	9	48			
**Recurrent bacteremia^b^**					
Yes	6 (24)	42 (11)	2.62	0.99–6.94	.05
No	19 (76)	349 (89)	Reference		
**ICU admission after BSI**					
Yes	7 (28)	88 (23)	1.34	0.54–3.31	.53
No	18 (72)	303 (77)	Reference		
**Intubated after BSI**					
Yes	4 (16)	55 (14)	1.16	0.38–3.52	.79
No	21 (84)	331 (86)	Reference		
Missing	0	7			
**Metastatic infection^c^**					
Yes	10 (40)	74 (19)	2.86	1.23–6.61	.01
No	15 (60)	317 (81)	Reference		
**Endocarditis**					
Yes	5 (50)	32 (43)	1.31	0.35–4.92	.69
No	5 (50)	42 (57)			
Missing	15	317			
**Days from BSI to discharge, median (IQR)**	34 (15–47)	15 (8–24)	1.55	0.97–2.49	.07

Note. MRSA, methicillin-resistant *Staphylococcus aureus*; BSI, bloodstream infection; ICU, intensive care unit; IQR, interquartile range

a Excluding patients on dialysis

b Positive blood culture over 30 days after the last positive blood culture

c Distal or secondary infection, anatomically unrelated to the primary site of infection, presenting during index admission of MRSA BSI. Includes infective endocarditis, septic pulmonary emboli, vertebral osteomyelitis, septic arthritis, iliopsoas abscess.

All 25 cases were initially treated with vancomycin monotherapy, and 7 patients (28%) completed treatment with vancomycin despite the elevated MIC because clinical response was achieved. For the remaining 18 patients (72%) who were switched from vancomycin, several reasons were documented: increasing MIC (n = 4), failure to clear cultures (n = 5), synergy of combination therapy (n = 5), persistent fevers (n = 1), difficulty dosing (n = 1), and lost parenteral access (n = 2). In 4 cases, regimens were adjusted due to detection of heterogeneous vancomycin-intermediate *S. aureus* (hVISA), but hVISA testing was stopped in 2016 and no longer influenced decision making.

Antibiotics utilized for the 18 cases (72%) that were switched from vancomycin therapy varied. Monotherapy was adjusted to daptomycin (n = 10), linezolid (n = 4), or telavancin (n = 1). Combination regimens included use of β-lactams (daptomycin-nafcillin, daptomycin-cefazolin, vancomycin-cefazolin, linezolid-cefazolin), and dual MRSA coverage (vancomycin-trimethoprim–sulfamethoxazole, vancomycin-daptomycin, linezolid-ceftaroline, vancomycin-ceftaroline, daptomycin-ceftaroline). Others included linezolid-tigecycline, and vancomycin-gentamicin. Adjunctive rifampin was used in 4 cases to clear bacteremia and reduce biofilm in disseminated infections. Three cases died or eloped prior to MIC elevation, and adjustments were not applicable.

## Discussion

Management of invasive *S. aureu*s is complicated by MRSA harboring elevated vancomycin MICs,^1^ which were present in 6% of the population with MRSA BSI in this study. Cases had higher odds of a personal history of MRSA colonization and hemodialysis, consistent with prior findings.^4^ Increased MRSA colonization in patients with frequent healthcare interactions has been established,^8^ and frequent healthcare interaction in turn increases risk of cumulative antibiotic exposure, especially vancomycin, which is a known cause of elevated MIC.^4^ Elevated vancomycin MIC was associated with metastatic infection but not increased mortality.

A survey of ID providers demonstrated variability in strategies for elevated vancomycin MICs.^5^ In our case series, rationales differed, often attributed to the elevated MIC or to failure to clear bacteremia. The most common alternative treatment was switching from vancomycin to daptomycin. Although salvage combination therapy was also utilized, this practice is based on case reports and series and requires randomized controlled studies.^9^ From the stewardship perspective, elevated MIC to vancomycin poses a unique challenge because, while susceptible, it prompts providers to adjust therapeutics. Minejima et al^10^ did not find antibiotic choice a significant factor in duration of bacteremia or outcome, and changing antibiotics may have unintended consequences of increased antibiotic class exposures, leading to potential drug resistance and toxicities without clear benefit.

Because MICs may vary between testing platforms, we selected a stringent MIC cutoff for case identification (MIC ≥ 2 µg/mL) to minimize testing variability, although we may have missed isolates for lack of serial MIC testing. Single-center, retrospective series are predisposed to inaccuracies from chart abstraction. Although all study patients received vancomycin, total vancomycin days were difficult to determine; we could not account for prior vancomycin exposure or antibiotic discontinuation in conjunction with palliation. Lastly, it is challenging to determine directionality when there is increased MIC and metastatic complications, where the metastatic infection may be a risk factor for the elevated vancomycin MIC.
